# Prevalence of Ear-Related Problems in Individuals Recovered From COVID-19

**DOI:** 10.22038/IJORL.2024.71040.3414

**Published:** 2024-05

**Authors:** Ahmadreza Nazeri, Amir Majidpour, Ali Nazeri, Ali Kamrani, Hashir Aazh

**Affiliations:** 1 *Department of Audiology, School of Rehabilitation, Shahid Beheshti University of Medical Sciences, Tehran, Iran.*; 2 *School of Medicine, Tehran University of Medical Sciences, Tehran, Iran.*; 3 *Department of Occupational Therapy, University of Social Welfare and Rehabilitation Sciences, Tehran, Iran. *; 4 *Hashir International Specialist Clinics & Research Institute for Misophonia, Tinnitus and Hyperacusis, London, UK.*

**Keywords:** Balance, COVID-19, Hearing loss, Hyperacusis, Tinnitus

## Abstract

**Introduction:**

The aim was to assess prevalence of tinnitus, hyperacusis, hearing and balance problems among patients recovered from COVID-19 infection. Self-reported ear and hearing symptoms were compared in three groups comprising: confirmed COVID-19, possible COVID-19, and non-COVID-19.

**Materials and Methods:**

1649 participants completed the survey in this cross-sectional study. The mean age was 34 years and 65% were female. Participants with confirmed and possible COVID-19 were asked if after their infection (compared to the past) they experienced hearing loss, ringing or whistling noises, fullness or blockage in their ears, loudness of the sounds that are normal to other people bother them more (an indication of hyperacusis), dizziness, giddiness, or imbalance.

**Results:**

Among participants with confirmed COVID-19, 16% reported that compared to the past their hearing has decreased, 21.5% noticed tinnitus, 22.5% aural fullness, 26.1% hyperacusis and 17.3% balance problems. Regression models showed that compared to the non-COVID-19 group, participants with confirmed COVID-19 had odds ratios (ORs) of significantly greater than 1 in predicting presence of self-reported symptoms of hearing loss, tinnitus, aural fullness, hyperacusis and balance problems, OR=1.96 (*p*=0.001), OR=1.63 (*p*=0.003), OR=1.8 (*p*<0.001), OR=2.2 (*p*<0.001), and OR=2.99 (*p*<0.001), respectively.

**Conclusions:**

There seem to be higher prevalence of self-report symptoms of ear-related problems among individuals with confirmed COVID-19 infection compared to a non-COVID-19 group during the pandemic.

## Introduction

A systematic review and meta-analysis examined 56 studies to assess the impact of COVID-19 on hearing and balance systems. They reported that the pooled estimated of prevalence of hearing loss, tinnitus and rotatory vertigo among patients with COVID-19 was 7.6%, 14.8% and 7.2%, respectively (1).

However, according to the authors of the systematic review, it was not clear if the cause of audio-vestibular symptoms was COVID-19 infection or the pandemic. A more recent systematic review assessed studies that have been published up to March 2021 regarding tinnitus and COVID-19 (2). They also concluded that although pooled estimated prevalence of tinnitus post COVID-19 was 8% (CI: 5 to 13%), there was no reliable evidence suggesting that tinnitus was caused by the COVID-19 infection as opposed to the general impact of the pandemic. Several research studies have been published since these systematic reviews with conflicting results. For example, Aazh, Danesh (3) compared the data with regard to tinnitus loudness, tinnitus annoyance and effect of tinnitus on life for patients with no symptoms of COVID-19 who were seen in their center during the COVID-19 lockdown and patients seen in the same period of the previous year, prior to the COVID-19 pandemic. Their results showed that the mean scores for tinnitus loudness, annoyance, and effect on life as measured via visual analogue scale did not differ significantly for the groups seen prior to and during lockdown. Consistent with this, Fioretti, Natalini (4) reported that among patients with tinnitus (95.5% of them did not have COVID-19 infection), tinnitus severity as measured via tinnitus handicap inventory (THI) (5) did not change significantly before and during the pandemic. Therefore, it seems that the pandemic itself is unlikely to have impacted on tinnitus at least among patients who did not experience COVID-19 infection. However, other studies on people who were infected with COVID-19 observed an impact on tinnitus and other hearing-related problems. For example, Thrane, Britze assessed hearing and tinnitus among 225 patients with COVID-19 symptoms (6). Their results showed that about 11% of participants reported hearing loss and 16.4% reported tinnitus which they believed were started on average 10 and 30 days, respectively, after onset of initial COVID-19 symptoms. Kartal and Kılıç (7) conducted a study on 279 individuals aged 18-60 years, who recovered from COVID-19 within the last month and did not have a chronic disease. About 10% (28/279) of them stated that they had no tinnitus before COVID-19 and that tinnitus started with COVID treatment and 6% (16/279) stated that tinnitus started after recovery from COVID-19. In another study, Verma, Shah (8) conducted a survey among people who recovered from COVID-19. They reported that about 5% of patients (4/78) had hearing related problems 15 days after COVID-19 recovery. Alves de Sousa, Pinto Costa (9) also reported COVID-19 impact by assessing audiometric thresholds among patients with COVID-19 without prior history of hearing problem and a matched control group. They reported that pure tone average (PTA) was about 25 dB HL in patients who had COVID-19 which was worse than PTA of 20 dB HL in controls (*p* = 0.001). To sum up, majority of the studies on individuals who did not have COVID-19 seem to suggest that the pandemic did not impact on tinnitus. However, studies on patients who experienced COVID-19 showed deterioration of hearing and tinnitus symptoms. As reviewed earlier, most studies on patients with COVID-19 had small sample sizes and/or did not distinguish COVID-19 confirmed with diagnostics tests from COVID-19 symptoms (i.e., possible COVID-19). To better understand the impact of COVID-19, it is important to distinguish confirmed COVID-19 from possible COVID-19. The aim of the present study is to assess self-report tinnitus, hyperacusis, hearing and balance problems among three groups comprising: (1) confirmed COVID-19 (tested positive) (2), possible COVID-19 (symptoms presented but they did not take any test), and (3) non- COVID-19. 

## Materials and Methods

*Ethical approval*

The Ethics Committee of Shahid Beheshti University of Medical Sciences (SBUMS), Tehran, Iran, approved the study protocol (IR.SBMU.RETECH.REC.1400.361).

*Study Design*

This was a cross sectional survey study to evaluate the effects of the COVID-19 disease on hearing and balance senses using a questionnaire. 

*Questionnaire Development*

The survey questionnaire (in Farsi) was designed in the Audiology Department of Rehabilitation Faculty of SBUMS, Tehran, Iran. Several specialists from audiology, ear-nose-throat (ENT), and occupational medicine met to discuss experiences with patients diagnosed with COVID-19. In each meeting, the providers analyzed diagnoses and complications related to sensory function in patients with COVID-19 and those who had recovered. At the end of these sessions, 14 items were developed. Following further discussions, five items were selected to remain on the final version of the questionnaire covering decreased hearing, tinnitus, aural fullness, hyperacusis, and balance problems. The questionnaire also collected information about the participants’ COVID-19 status and demographic information comprising age, gender, and ethnicity.

Formal psychometric tests deemed unnecessary as this questionnaire did not measure any specific construct. This questionnaire was intended to act as a simple checklist for gathering facts about the participant’s’ COVID-19 diagnosis and assess if they report any ear- and hearing-related symptoms with yes/no answers. Each item was scored separately and there was no total score for the questionnaire. This initial version of the questionnaire was used for a pilot study on 50 people (23 recovered from COVID-19 and 27 non-COVID-19 controls) selected by purposeful sampling (10). After reviewing the results, resolving possible defects, and applying the participants’ viewpoints about the questions, the final form of the questionnaire was prepared to be utilized in the survey.

*Questionnaire Structure*

*Section1:* Demographic information comprising age, gender, and ethnicity.

*Section 2:* This section was used to establish COVID-19 status. Participants were asked if they have COVID-19 since the onset of the outbreak? If they answered “no” then they were directed to Section 3. If they answered “yes” then they were asked further questions comprising: (1) When was the definitive diagnosis date of your COVID-19? (2) How was the positive result of COVID-19 diagnosed? (PCR (polymerase chain reaction) test, antibody test, CT scan (Computed tomography) or X Ray) (3), How was your treatment and recovery done? (4) Were you hospitalized? (5) Did you have any underlying disease before you got infected with the COVID-19? The option choices for pre-existing illnesses were diabetes, high blood pressure, heart disease, respiratory discomfort, rheumatic (or any autoimmune diseases), under treatment with oral or injectable corticosteroids, liver and kidney failure, other diseases, and no known underlying disease. Participants could choose more than one option.

Participants were categorized into three groups based on their responses to the COVID-19 questions comprising: (1) confirmed COVID-19 (diagnosis was made based on either PCR test, antibody test, or CT scan/X Ray), (2) possible COVID-19 (reported experiencing COVID-19 symptoms but they did not take any test to confirm it), and (3) non-COVID-19 (no COVID-19 symptoms were reported). 

*Section 3:* Participants with confirmed and possible COVID-19 were asked if after their infection (compared to the past) they experienced (1) decreased hearing (yes/no) (2), ringing, buzzing or whistling noises in their ears (yes/no) (3), fullness or blockage in their ears (yes/no) (4), loudness of the sounds that are normal for other people bother them more [an indication of hyperacusis] (yes/no) (5), dizziness, giddiness, or imbalance (yes/no). Participants with no COVID-19 were asked to answer each of these questions based on how they felt since the onset of COVID-19 pandemic. 

*Study Population and Data Collection*

The sampling period was from 28^th^ February to 27^th^ April, 2021. In addition to hard copies, the online form of the questionnaire was utilized through the Porsline website (https://porsline.ir/), and the internet link was provided to the participants to complete the questionnaire. A total of 95% of the questionnaires were distributed via Telegram social media. The questionnaire was distributed throughout Iran via social networks in order to cover the majority of ethnicities. The questionnaire link was also advertised on a Telegram channel with over 1,500,000 public subscribers that broadcast daily Persian news. People living in Iran of age of ten years, or more were invited to complete the survey. The text of the invitation was as follows: "This questionnaire was designed by Shahid Beheshti University of Medical Sciences in order to understand the complications of COVID-19 better. Thank you in advance for taking the time to complete this questionnaire." The participants were required to read a page outlining the study and then check a box indicating their desire to participate. Participants were also made aware that they could withdraw from the study at any time if they wished. After providing consent, participants completed the questionnaire. During the study period, 2590 people visited the online questionnaire, of whom 1522 individuals completed the questionnaire. An additional 127 individuals completed the printed version of the same questionnaire which were distributed by convenience sampling (11) among the people who were referred to the Tehran’s audiology screening center. 

*Data Analysis*

The descriptive data were reported in the form of percentages, means, and standard deviations (SD). Chi-squared (^2^) tests were used to compare the proportions of pre-existing illnesses between participants with confirmed and possible COVID-19. Independent-samples *t*-tests and one-way analysis of variance (ANOVA) were used to compare mean age among different groups. 

Based on participants’ responses five variables were created for ear-related problems comprising: tinnitus (yes/no), hearing loss (yes/no), aural fullness (yes/no), hyperacusis (yes/no), and balance problems (yes/no). 

Kruskal-Wallis test (one-way ANOVA on ranks) (12) was used to assess if there was a statistically significant difference in proportions of ear-related problems among the groups. Logistic regression was performed to assess whether participants’ groups based on their COVID-19 diagnosis (independent variables) is related to the presence or absence of ear-related problems (dependent variables). Odds ratios (ORs) and their 95% confidence intervals (95% CI), adjusted for age and gender, were calculated separately for each of the ear-related problems comprising: (1) hearing loss, (2) tinnitus, (3) aural fullness, (4) hyperacusis, and (5) balance problems. The participants’ groups included in each model comprised (1) confirm COVID-19, (2) possible COVID-19, and (3) non-COVID-19. The prevalence of COVID-19 infection in Iran in August 2020 was estimated as 14.2% of the population over 6 years of age (11 958 346 people) (13). The prevalence in densely populated cities and provinces on average was estimated to be over 17% with some cities reaching to approximately 57% (14,15). Based on these studies, we used an average of 30% prevalence of COVID-19 in Iran to estimate sample size for the current study. A sample size of above 857 participants gives a desired precision of 0.03 with confidence level of 0.95. 

The *p *value required for statistical significance was *p* <0.05. The number of participants included in each analysis (*n*) is reported. SPSS software (version 23.0) (16) was used for statistical analysis. 

## Results

*Participants and Their COVID-19 Status*

The total number of completed questionnaires was 1649. The mean age for the participants was 34 years (SD=11.3) ranging from 10 to 95 years old. Sixty five percent of participants (1066 out of 1649) were female. Majority of participants (68.5%) identified themselves as Persians and this was followed by 11.2% as Kurds, 9.9% Turks, 4.3% Lors, 3% Gilaks, 2.4% others, and 0.7% Laks. 

28.8% of the participants (475/1649) had confirmed COVID-19, among which 319 had positive PCR test, 173 were diagnosed based on CT scan/X ray, and 73 based on an antibody test (some participants had more than one diagnostic test). Among the participants with confirmed COVID-19, 82.9% had recovered after being quarantined at home, 2.8% had been hospitalized, and 14.3% continued their routine lives despite being diagnosed with COVID-19 disease. 39.3% of participants (648/1649) were categorized into possible COVID-19 group. It was impossible to know if they had COVID-19 or not as they did not take a test. Finally, 31.9% (526/1649) who did not report symptoms of COVID-19 hence were categorized to the non-COVID-19 group. 

*Comparison of Pre-existing Illnesses Between Confirmed COVID-19 and Possible COVID-19*

Table 1 compares the proportion of the pre-existing illnesses between the participants with confirmed COVID-19 and possible COVID-19. Compared with possible COVID-19, participants with confirmed COVID-19 had higher proportions of pre-existing respiratory illness, hypertension, heart disease, and liver and kidney failure (*p*<0.05). Respiratory illness followed by hypertension were the most prevalent pre-existing conditions in this group with prevalence of 6.1% and 5.7%, respectively. Unfortunately, the data regarding the pre-existing illnesses was not collected for participants in non-COVID-19 group.

**Table 1:** The prevalence of pre-existing illnesses between confirmed and possible COVID-19 cases using the Kruskal-Wallis test

**Pre-exiting illnesses**	**Confirmed COVID-19** **(** ***n*** **=475)**	**Possible COVID-19 ** **(** ***n*** **=648)**	^2^	***p*** **-value**
Diabetes	2.7% (*n*=13)	1.2% (*n*=8)	3.4	0.06
Hypertension	5.7% (*n*=27)	1.4% (*n*=9)	16.3	<0.001
Respiratory illness	6.1% (*n*=29)	0.9% (*n*=6)	24.3	<0.001
Heart disease	2.3% (*n*=11)	0.6% (*n*=4)	6.0	0.014
Rheumatic or autoimmune diseases under treatment	2.1% (*n*=10)	0.8% (*n*=5)	3.7	0.054
Under treatment with oral or injectable corticosteroids	1.1% (*n*=5)	0.3% (*n*=2)	2.4	0.12
Liver or kidney failure	1.5% (*n*=7)	0	9.6	0.002
Other pre-existing diseases	4.8% (*n*=23)	1.5% (*n*=10)	10.5	0.0012
None	78.5% (*n*=373)	94.6% (*n*=613)	143.5	<0.001

*Comparison of Age and Gender Distribution Among the Groups*

Mean age was 35.5 years (SD=10.5) for participants with confirmed COVID-19 compared to 32.2 years (SD=10.7) for participants in possible COVID-19 group, and 35.7 years (SD=12.5) for non-COVID-19 group. On average, the participants in non-COVID-19 group and confirmed COVID-19 were older than the participants in possible COVID-19 group (*F* (2, 1646) =17.6, *p*<0.001). Gender distribution (female/male) was 289/186 for participants with confirmed COVID-19, 442/206 for possible COVID-19, and 335/191 for non-COVID-19 

group ( ^2^ = 6.8, *p *= 0.033). As shown in Table 2, 50.7% of participants with confirmed COVID-19 reported at least one ear-related problem compared to 43.8% of participants with possible COVID-19 and 32.5% of the non-COVID-19 group (*p*<0.001) using Kruskal Wallis Test. 

Based on participants responses to the survey questions, self-report symptoms of hearing loss, tinnitus, ear fullness, hyperacusis and balance problems were more prevalent among participants in confirmed COVID-19 group compared to possible COVID-19 and the non-COVID-19 groups.

**Table 2:** Comparison of ear-related problems in confirmed COVID-19, possible COVID-19, and non-COVID-19 groups using the Kruskal-Wallis test

**Ear-related problems**	**ConfirmedCOVID-19 ** ***(n*** **=475)**	**Possible ** **COVID-19(** ***n*** **=648)**	**Non-COVID-19** ***(n*** **=526)**	^2 ^**(degree of freedom)**	***p*** **-value**
Hearing Loss	16% (*n*=76)	13.1% (*n*=85)	8.9% (*n*=47)	11.5 (2)	0.003
Tinnitus	21.5% (*n*=102)	17.7% (*n*=115)	14.4% (*n*=76)	8.4 (2)	0.015
Aural fullness	22.5% (*n*=107)	19.9% (*n*=129)	13.9% (*n*=73)	13.2 (2)	0.0014
Hyperacusis	26.1% (*n*=124)	19.6% (*n*=127)	14.3% (*n*=75)	22.1 (2)	<0.0001
Balance problems	17.3% (*n*=82)	11.7% (*n*=76)	6.7% (*n*=35)	27.2 (2)	<0.0001
No reported problem	49.3% (*n*=234)	56.2% (*n*=364)	67.5% (*n*=355)	35.1 (2)	<0.0001

Logistics regression models showed that compared to the non-COVID-19 group, adjusted for age and gender, participants with confirmed COVID-19 had ORs of significantly greater than 1 in predicting presence of hearing loss, tinnitus, aural fullness, hyperacusis and balance problems (Table.3). The OR for balance problems in confirmed COVID-19 cases compared to the non-COVID-19 group was the largest (OR=2.99 (95% CI: 1.96, 4.54, *p*<0.001). Participants in possible COVID-19 group also had ORs of significantly greater than 1 in predicting presence of hearing loss, aural fullness, and balance problems. However, possible COVID-19 did not significantly predict presence of tinnitus and hyperacusis with non-COVID-19 group as the reference (OR=1.22 (95% CI: 0.89, 1.69, *p*=0.2) and OR=1.36 (95% CI: 0.99, 1.86, *p*=0.06), respectively).

**Table 3:** Logistic regression models reveal odds ratios for ear-related issues in confirmed and unconfirmed COVID-19 groups compared to a non-COVID-19 group

**Ear-related problems**	**Groups**	**Adjusted Odds Ratio (95% Confidence Intervals)**	***p*** **-value**
Hearing Loss	Non-COVID-19	1	
	Unconfirmed COVID-19	1.62 (1.11, 2.37)	0.013
	Confirmed COVID-19	1.96 (1.33, 2.89)	0.001
Tinnitus	Non-COVID-19	1	
	Unconfirmed COVID-19	1.22 (0.89, 1.69)	0.2
	Confirmed COVID-19	1.63 (1.17, 2.26)	0.003
Aural fullness	Non-COVID-19	1	
	Unconfirmed COVID-19	1.55 (1.13, 2.13)	0.006
	Confirmed COVID-19	1.8 (1.30, 2.51)	<0.001
Hyperacusis	Non-COVID-19 group	1	
	Unconfirmed COVID-19	1.36 (0.99, 1.86)	0.06
	Confirmed COVID-19	2.2 (1.59, 3.05)	<0.001
Balance Problems	Non-COVID-19	1	
	Unconfirmed COVID-19	1.79 (1.17, 2.73)	0.007
	Confirmed COVID-19	2.99 (1.96, 4.54)	<0.001

## Discussion

Our results showed that over 50% of participants with confirmed COVID-19 reported to have at least one ear-related problem. This is significantly higher than what was observed among the possible COVID-19 and the non-COVID-19 controls groups. There are two possible explanations for this comprising: (1) the participants with possible COVID-19 might have been experiencing COVID-19 or other illnesses which resemble the symptoms of COVID-19 such as influenza, common cold or SARS (severe acute respiratory syndrome). It is plausible that the impact of COVID-19 on hearing and balance organs could be more severe than that of influenza, common cold, and SARS (2,17,18). Second possible explanation is that certain pre-existing illnesses (i.e., respiratory illness, hypertension, heart disease, and liver and kidney failure) were more prevalent in the confirmed COVID-19 group compared to the possible COVID-19 group. This might have influenced the observed differences in ear-related problems between confirmed and possible COVID-19 groups. For example, past studies suggest that individuals with diabetes have twice the incidence of hearing loss (19) and the medications used for treatment of certain respiratory infections are ototoxic and cause hearing loss and tinnitus (20). Hypertension, cardiovascular abnormalities, and chronic kidney disease have also been identified as possible risk factors for tinnitus and sensorineural hearing loss (21-23). 

Unfortunately, our sample size was not large enough to adjust the analyses based on the pre-existing illnesses. In addition, due to a design limitation in the survey questionnaire, the patients in the non-COVID-19 group were not asked about their pre-existing illnesses making it impossible to take this into account in the analysis. Future studies should assess pre-existing illnesses among patients with and without COVID-19. A systematic review and meta-analysis reported that the prevalence of hearing loss and tinnitus was higher than rotatory vertigo among patients with COVID-19 (1). Our results suggests that COVID-19 increases the risk of hearing loss, tinnitus, hyperacusis, and aural fullness by about 1.9 times on average, however it increases the risk of balance problems by a factor of 3. Therefore, based on our data, there was a stronger association between COVID-19 and balance problems compared to ear-related problems. This observation may be related to the fact that we used a more generic description for balance problems (i.e., dizziness, giddiness, or imbalance) compared to focusing on rotatory vertigo as was done in the systematic review conducted by Almufarrij and Munro (1). Consistent with this, a more recent meta-analysis and systematic review which included dizziness, rotatory vertigo and vestibular dysfunction to their analysis reported that the prevalence of balance problems was approximately 3 times the prevalence of hearing loss or tinnitus in patients with COVID-19 (24). 

*Study limitations*

The study sample may not be representative of the general population in Iran as most participants were recruited online via social media. Therefore, interpreting results should be with caution. Participants were requested to disclose the date of their positive test outcome and specify the type(s) of test undertaken. Due to the survey's structure, we lacked the means to independently validate these test results, thus constituting a limitation of our study. Nevertheless, it's important to note that standard diagnostic procedures employed in Iran during the pandemic included PCR, antibody tests, CT scans, and X-rays. CR tests are recognized for their reliability in detecting viral RNA through reverse transcription polymerase chain reaction (25). Serological tests, or antibody tests, identify antibodies generated in response to COVID-19. (26). Additionally, CT scans and chest X-rays are frequently utilized as primary diagnostic tools for COVID-19 detection. (27-30). Another constraint of this investigation involves depending on participants' self-reported symptoms, which could have been susceptible to various forms of bias (31-33). For example, the very fact that respondents were being asked to judge if they experienced hearing problems or tinnitus after their infection (compared to the past) might have led to them believe that there were expected to be such effects, and to respond accordingly. In future studies, efforts should be made to overcome the limitations of our investigation. Firstly, it would be beneficial to diversify the recruitment methods to ensure a more representative sample of the Iranian population. While online recruitment via social media platforms offers convenience and accessibility, it may introduce limit the generalizability of findings. Therefore, employing additional recruitment strategies such as community outreach programs or healthcare facilities could enhance the sample's diversity and representativeness.

Secondly, the validation of participants' self-reported COVID-19 test outcomes is crucial to ensure the accuracy and reliability of data. Future research endeavors should incorporate methods for independently verifying test results through medical records or laboratory documentation. This validation process can help mitigate the potential inaccuracies associated with self-reported test outcomes and enhance the credibility of study findings.

In conclusion, future studies should address the aforementioned limitations and adopt a multidisciplinary approach to investigate the hearing manifestations of COVID-19 comprehensively. By employing diverse recruitment methods, validating test outcomes, utilizing comprehensive diagnostic procedures, and employing standardized assessment tools, researchers can enhance the quality and reliability of data and contribute to a more thorough understanding of the hearing sequelae of COVID-19 infection.

## Conclusion

Compared to non-infected individuals, people with confirmed or possible COVID-19 reported higher proportion of hearing loss, tinnitus, aural fullness, hyperacusis and balance problems. Both possible and confirmed COVID-19 cases had ORs of significantly greater than 1 in predicting hearing loss, aural fullness, and balance problems. Unlike possible COVID-19 which did not predict tinnitus and hyperacusis, confirmed COVID-19 was significantly associated with presence of tinnitus and hyperacusis compared to the non-COVID-19 group. However, this study relied on participants’ self-reported recall of their symptoms without using clinical investigations. Therefore, future studies should compare the results of audio-vestibular investigations between patients with COVID-19 infection and a control group. 
